# Evidence for glucocorticoid-mediated hypertension after uninephrectomy

**DOI:** 10.1002/phy2.101

**Published:** 2013-10-11

**Authors:** Carina Huesler, Meret Lauterburg, Brigitte M Frey, Felix J Frey

**Affiliations:** 1 Department of Nephrology, Hypertension and Clinical Pharmacology, University Hospital BerneBerne, Switzerland; 2 Department of Clinical Research, University Hospital BerneBerne, Switzerland

**Keywords:** 11β-hydroxysteroid, dehydrogenase, hypertension, kidney tubules, salt sensitivity

## Abstract

Recently, evidence was presented that uninephrectomy induces salt-sensitive hypertension in rats. The increase in blood pressure was abrogated by a mineralocorticoid receptor antagonist but not by an aldosterone synthase inhibitor. Here, we hypothesize that mineralocorticoid receptor activation occurred by an 11beta-hydroxy-glucocorticosteroid, as a consequence of dysregulated 11beta-hydroxysteroid dehydrogenase enzymes. Therefore, 3-week-old Sprague-Dawley rats were either uninephrectomized or sham operated and given a normal (0.4%) or high (8%)-salt diet. At week 8, a telemetric device was implanted, and during week 13 blood pressure continuously measured and urine was collected. The animals were sacrificed thereafter and liver and kidney were harvested. Steroid metabolites were analyzed by GC-MS and mRNA assessed by PCR. Uninephrectomy caused a distinct salt-sensitive hypertension. The increase in blood pressure correlated significantly with a decline in the apparent activity of 11beta-hydroxysteroid dehydrogenase 2 and an increase of 11beta-hydroxysteroid dehydrogenase 1, when urinary corticosterone metabolites were considered. These results were mirrored by the corresponding metabolite ratios assessed in renal and liver tissue. Changes in enzyme activities were in part explained by changes in the mRNA content. In conclusion, mineralocorticoid receptor-dependent salt sensitivity after UNX in rats appears to be mediated by glucocorticoids.

## Introduction

The kidneys play a pivotal role in blood pressure control. A reduced nephron number is a risk factor for arterial hypertension (Hughson et al. 2003; Keller et al. 2003; Zohdi et al. 2012). Not surprisingly, humans with only one kidney are prone to develop arterial hypertension. Although uninephrectomy (UNX) for renal transplantation is considered to have minimal adverse effects on overall health status, a meta-analysis revealed an increase in blood pressure of 5 mmHg over 5–10 years beyond that associated with normal aging (Boudville et al. 2006; Ibrahim et al. 2009). Similarly, children with one missing kidney at birth, more frequently develop arterial hypertension than children with two kidneys (Westland et al. 2011). The relevance of two kidneys for normal tension is although backed by investigations in rats (Woods et al. 2001; Carlström et al. 2007). Recently, Kawarazaki et al. (2011) demonstrated that the effect of UNX on blood pressure is age dependent. When these investigators removed the kidney at week 3, a more pronounced increase in blood pressure and salt sensitivity was observed than when the operation was performed at week 10. The increase in blood pressure was mineralocorticoid receptor (MR) dependent, as shown by treating the rats with the MR-antagonist eplerenone. Interestingly, the aldosterone concentrations in UNX rats on high salt (HS) were suppressed, suggesting that aldosterone was not the culprit for MR activation, a conclusion supported by the observation that the administration of the aldosterone synthase inhibitor FAD286 did not abrogate the increase of blood pressure in the Sprague-Dawley rats after UNX under a HS diet. Thus, another mechanism of MR activation than aldosterone has to be considered for the induction of hypertension after UNX. Here, we present evidence for a glucocorticoid-mediated MR activation.

**Figure 1:**
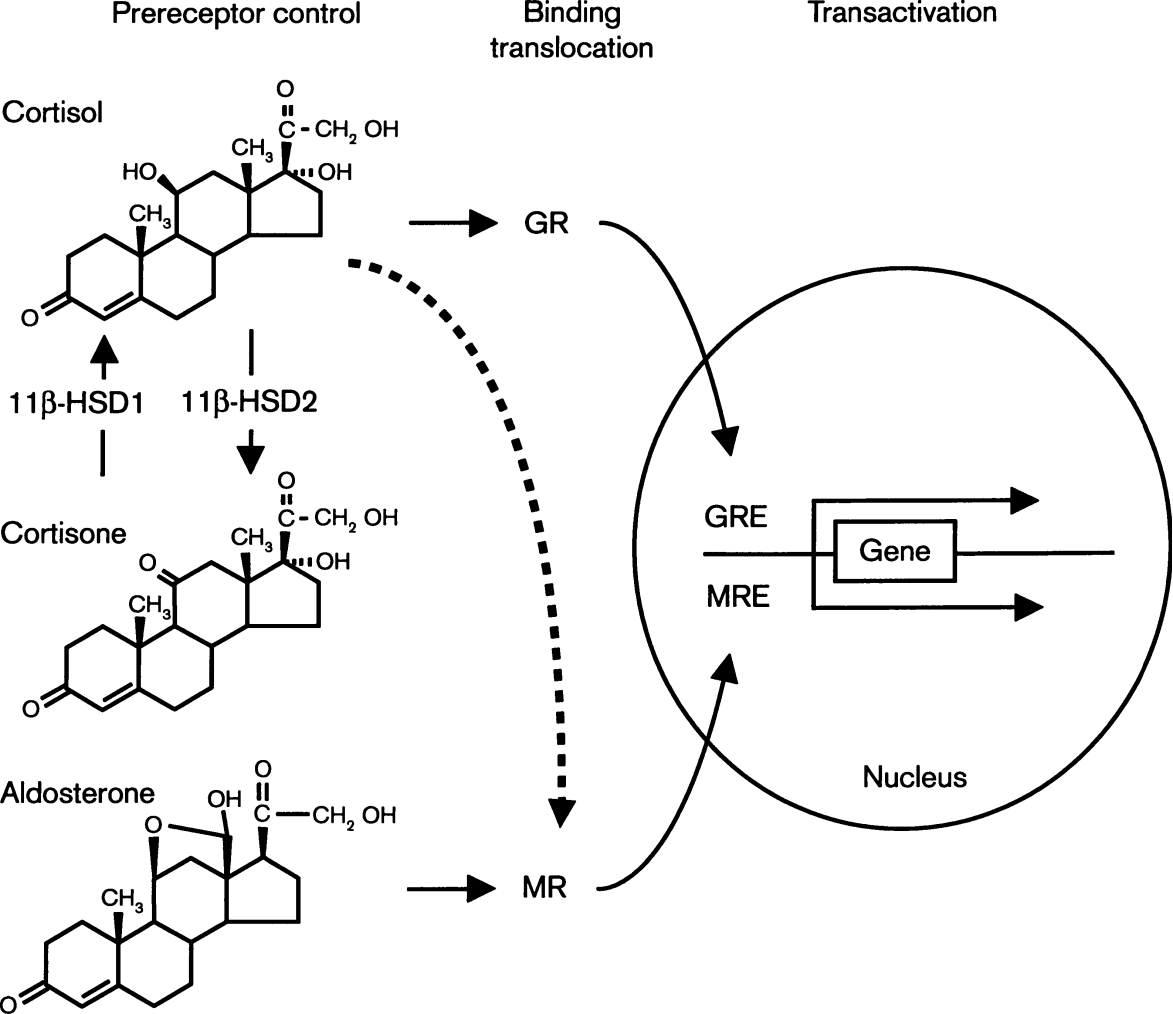
Glucocorticoid receptors and mineralocorticoid receptors are ligand-inducible transcription factors. Following binding of steroid hormones, these receptors translocate from the cytoplasm into the nucleus and display their transactivation potential. The intracellular concentrations of steroid molecules available for binding to the cognate receptor depends on the free extracellular concentrations and an intracellular prereceptor control mechanism constituted by the 11βHSD1 and 2 enzymes. Whereas 11βHSD1 acts predominantly as reductase and converts the 11-keto-steroid cortisone with virtually no affinity for mineralocorticoid receptors and glucocorticoid receptors into the 11β-hydroxy-glucocorticoid cortisol with a high affinity for both glucocorticoid receptors and mineralocorticoid receptors, 11βHSD2 is exclusively an oxidase and inactivates cortisol into cortisone, which allows protection of mineralocorticoid receptor-expressing cells from promiscuous activation of mineralocorticoid receptors by the glucocorticoid hormone cortisol. GRE, glucocorticoid-response element, MRE, mineralocorticoid-response element (Frey et al. 2004).

The corticosteroid aldosterone has a high affinity for the MR and a lower affinity for the glucocorticoid receptor (GR) (Edwards et al. 1989; Frey et al. 2004). Conversely, cortisol in humans and corticosterone in rodents have a high affinity for the GR and are thus considered to be the endogenous glucocorticoids. These two glucocorticoids, however, have the same affinity for the MR as aldosterone and are present in higher concentrations by several orders of magnitude. Nevertheless, aldosterone appeared to account for the main MR effects in vivo. In 1988, Funder et al.^1988^ and Edwards et al.^1988^ proposed that a prereceptor mechanism allows to inactivate corticosterone and cortisol in mineralocorticoid target tissues (Fig. 1). Thus, we hypothesize now that a dysregulation of this prereceptor mechanism, the 11β-hydroxysteroid dehydrogenase enzymes (11βHSD), causes a glucocorticoid-mediated MR activation after UNX in rats on a HS diet. For that purpose, we uninephrectomized rats at week 3, analyzed the 11βHSD activities on a normal (NS) or HS diet, and measured blood pressure by telemetry. The observations indicate an enhanced availability of glucocorticoids due to an increased 11βHSD1 and a diminished 11βHSD2.

## Methods

### Animals

All animal studies were done in accordance with the Felasa recommendations and were approved by the Ethical Committee for Animal Research of the University of Berne. Three-week-old male Sprague-Dawley rats (Charles River Laboratories, Sulzfeld, Germany) were assigned to a UNX or sham (Sham) operation and then exposed to either a HS (8%) or a NS (0.4%) diet for 10 weeks. In this way, the following four subgroups were analyzed: Sham-operated rats fed with a NS (NS-Sham) or a HS diet (HS-Sham) and uninephrectomized rats raised with a NS (NS-UNX) or a HS diet (HS-UNX). The pellets with variable salt content were prepared by Kliba AG, Kaiseraugst, Switzerland. Five weeks after UNX or sham surgery, a telemetric blood pressure device (Datascience International, St. Paul, MN) was implanted to measure blood pressure and heart rate. All animals were housed in groups in a room with constant temperature (22 ± 2°C), humidity (60 ± 5%), and a 12:12 h light-dark cycle. The animals received water and food ad libitum. In week 13, the rats were kept individually in metabolic cages for monitoring of food and water intake and collection of urine. During the week of isolation, blood pressure was measured for 10 sec every 15 min by telemetry while rats were conscious and moving freely. At the end of this week, rats were sacrificed, liver and kidney tissue as well as blood were harvested. Organs were frozen in liquid nitrogen and stored at −70°C. For quantification of mRNA, the tissues were powdered by mortar and pestle with a mixture of acetone and dry ice. Plasma was separated by centrifugation and stored at −20°C.

### Surgical procedures

Right-sided UNX as well as the sham surgery was performed at week 3 as described before (Schumacher et al. 2002). The implantation of the telemetric blood pressure device (PA-C40, DSI™; Transoma Medical, St Paul, MN) was performed in all, UNX and sham-operated, rats at week 8 as described previously (Schumacher et al. 2002).

### Measurement of aldosterone by ELISA

Plasma aldosterone was measured using an enzyme-linked immunosorbent assay kit for rat aldosterone (USCN Life Science Inc., Wuhan, China).

### Analysis of steroids by gas chromatography–mass spectrometry

The metabolites corticosterone (B), dehydrocorticosterone (A), tetrahydrocorticosterone (THB), 5-α-tetrahydrocorticosterone (5αTHB), and tetrahydrodehydrocorticosterone (THA) were analyzed in plasma, urine, kidney, and liver tissue by gas chromatography–mass spectrometry. A quantity of 100 μg of homogenized tissue was extracted like plasma samples, urine and plasma were extracted and analyzed as described before (Audige et al. 2002). The in vivo activities of HSD1 and HSD2 were assessed by the ratios of (THB + 5αTHB)/ THA and B/A, respectively.

### Quantification of 11βHSD1 and 2 mRNA in tissues

RNA extraction and quantification of HSD1 and 2 mRNA were performed as described previously (Lauterburg et al. 2012). Two microgram of RNA was reversely transcribed with AMV Reverse Transcriptase (Invitrogen, Basel, Switzerland) and 11βHSD1 and 11βHSD2 mRNA levels were analyzed by real-time PCR using standard conditions and appropriate assay on demand from applied biosystems (11β-OHSD1, Rn00567167_m1; 11β-OHSD2, Rn00492539_m1). GAPDH (4352338E) was used as internal standard.

### Statistical analysis

Data are expressed as mean ± SEM. Two-way ANOVA was used for comparisons between groups. Correlations were calculated by linear regression. All statistical tests were performed using GraphPad Prism version 5.00 for Windows (GraphPad Software, San Diego, CA, http://www.graphpad.com). *P* values of *P* < 0.05 were considered to indicate statistical significance.

## Results

### Mean arterial blood pressure and heart rate

**Figure 2:**
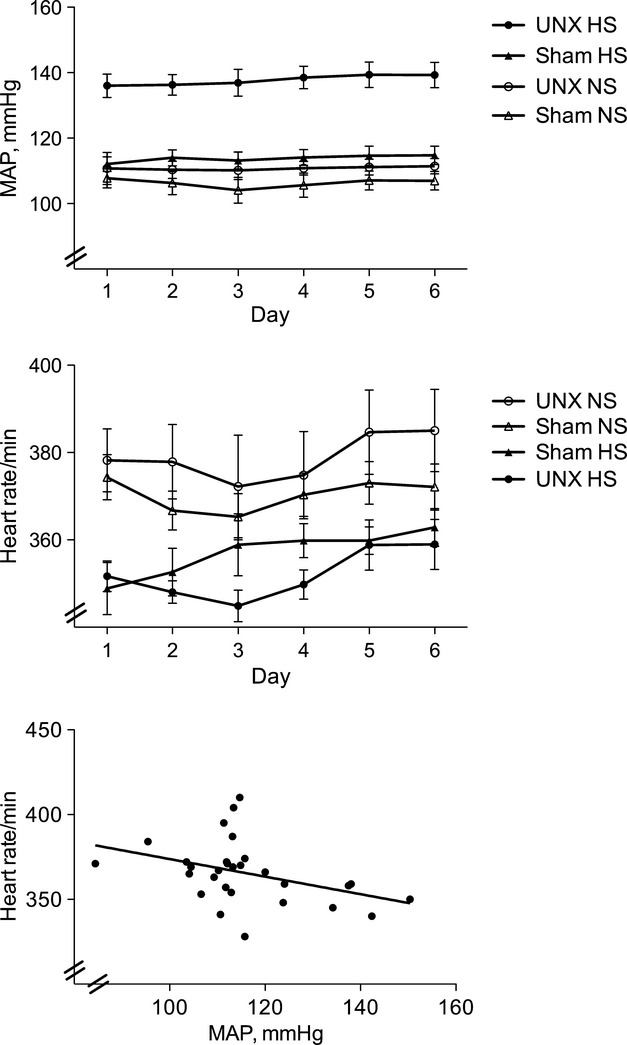
Effect of Uninephrectomy (UNX) and normal (NS) or high-salt (HS) diet on mean arterial blood pressure (MAP) and heart rate and relation between MAP and heart rate in Sprague-Dawley rats. The analysis by two-way ANOVA indicates that both UNX and HS diet independently increase MAP and decrease heart rate (*P* < 0.0001 for the two sources of variation and *P* = 0.0034 for interaction). For each animal the mean heart rate and MAP from day 1 to day 6 was calculated. When these values were considered as a group (*n* = 29), an inverse relationship between MAP and heart rate was observed (*r* = 0.39, *P* = 0.036). Sham indicates sham operated. Each group comprised 6–8 animals. Values are means ± SEM.

Uninephrectomy increased mean arterial blood pressure (MAP) in rats on a NS or a HS diet. Independent of UNX, rats on a HS diet had a higher MAP than rats on a NS diet. As a corollary, the combination of UNX and HS diet appears to enhance MAP most strikingly (HS-UNX: 137.8 ± 3.6 mmHg, HS-Sham: 113.8 ± 2.4 mmHg, NS-UNX: 110.8 ± 2.6 mmHg, NS-Sham: 106.3 ± 3.3mmHg; *P* < 0.0001, Fig. 2). Although, the effect of the interventions on heart rate was small, it appears to be consistent and inversely linked with MAP (HS-UNX: 352 ± 3.3/min, HS-Sham: 357.2 ± 3.9/min, NS-UNX: 378.9 ± 8.9/min, NS-Sham: 370.2 ± 4.2/min; *P* < 0.05 for the intervention factor salt diet, not significant for the intervention factor UNX, Fig. 2).

### Body weight and renal function

**Figure 3:**
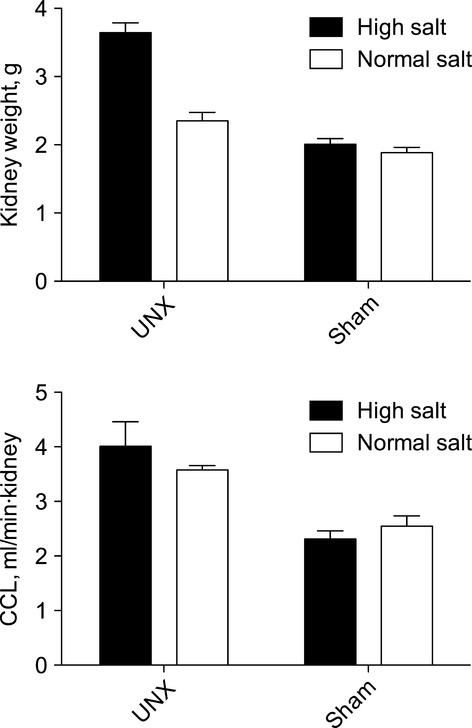
Impact of uninephrectomy (UNX) and normal (NS) or high-salt (HS) diet on kidney weight and creatinine clearance (CCL) in Sprague-Dawley rats. In UNX rats the right kidney was removed and the weight and CCL of the left kidney were considered. In sham operated (Sham) animals the weight of the left kidney was determined and the values of CCL divided by two. A two-way ANOVA analysis suggests that both UNX and high salt independently increase kidney weight (*P* < 0.0001 for the two sources of variation and for interaction), whereas only UNX and not salt diet enhanced CCL (*P* < 0.0001). Mean values (±SEM) are given for each group of animals (*n* = 7–9).

Uninephrectomy and/or different salt diets did not significantly modulate body weight of the rats (results not given). As expected, urine production and urinary sodium excretion increased several fold in rats on a HS diet (Table 1). The daily amount of sodium recovered in urine reflected the amount of salt administered to the various groups of rats investigated (results not given). No consistent impact of UNX on daily creatinine excretion was encountered (Table 1). Creatinine clearance per kidney and kidney weight increased after UNX, an effect enhanced by HS diet (Creatinine clearance per kidney: HS-UNX: 4.01 ± 0.45 mL/(min × kidney), NS-UNX: 3.58 ± 0.08 mL/(min × kidney), HS-Sham: 2.31 ± 0.30mL/(min × kidney), NS-Sham: 2.55 ± 0.38 mL/(min ×kidney); *P* < 0.0001 for intervention factor UNX; weight of the left kidney: HS-UNX: 3.65 ± 0.14 g, NS-UNX: 2.45 ± 0.08 g, HS-Sham: 2.01 ± 0.09 g, NS-Sham: 1.89 ± 0.08 g; *P* < 0.0001, Fig. 3).

**Table 1:** Biochemical data and statistics of uninephrectomized and sham-operated rats fed with a high- or normal salt diet

	Uninephrectomy		Sham operated		Two-way ANOVA		
			
	High salt, *n* = 7	Normal salt, *n* = 8	High salt, *n* = 8	Normal salt, *n* = 9	Salt diet	Uninephrectomy	Interaction
Urinary chemistry							
Volume, mL/day	68 ± 3.0	13 ± 1.5	65 ± 5.4	16 ± 1.4	*P* < 0.00001	n.s.	n.s.
Creatinine, μmol/day	128.4 ± 5.0	127.4 ± 3.0	111.9 ± 7.8	143.3 ± 6.4	n.s.	*P* < 0.01	*P* < 0.01
Sodium, mmol/L	425.1 ± 13.7	123.0 ± 13.3	435.9 ± 22.2	111.0 ± 10.1	*P* < 0.0001	n.s.	n.s.
Potassium, mmol/L	24.8 ± 1.2	109.2 ± 12.8	28.2 ± 3.3	100.0 ± 16.5	*P* < 0.00001	n.s.	n.s.
mRNA in tissue							
11β-HSD1 in liver/GAPDH	0.935 ± 0.09	0.766 ± 0.16	0.843 ± 0.10	0.464 ± 0.09	*P* < 0.01	n.s.	n.s.
11β-HSD2 in kidney/GAPDH	0.117 ± 0.02	0.075 ± 0.01	0.119 ± 0.01	0.112 ± 0.03	n.s.	n.s.	n.s.
Urinary catecholamines							
Dopamine, nmol/day	50.7 ± 5.7	48.3 ± 16.2	47.5 ± 5.0	91.2 ± 12.2	n.s.	n.s.	*P* < 0.01
Epinephrine, nmol/day	1.17 ± 0.24	1.58 ± 0.14	0.729 ± 0.27	2.35 ± 0.35	*P* < 0.001	n.s.	n.s.
Norepinephrine, nmol/day	17.0 ± 1.4	16.0 ± 2.3	20.2 ± 2.5	27.1 ± 3.1	n.s.	*P* < 0.001	n.s.

### Estimation of apparent activity of 11β-hydroxysteroid dehydrogenase types 1 and 2

**Figure 4:**
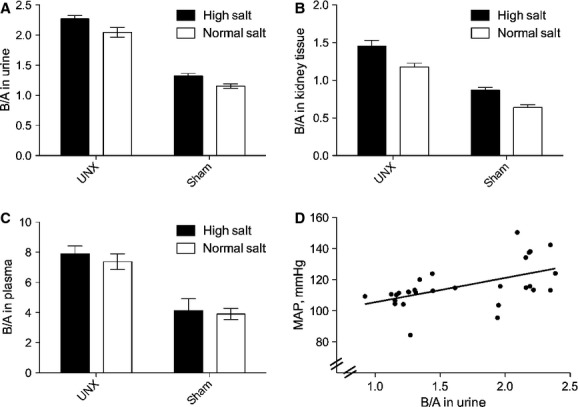
Comparison of Corticosterone/Dehydrocorticosterone (B/A) in urine (A), kidney tissue (B), and plasma (C) between uninephrectomized (UNX) and sham-operated (Sham) rats fed a normal or high-salt diet. The ANOVA revealed that UNX increased the steroid ratios in urine, kidney tissue, and plasma (*P* < 0.0001). A high-salt diet enhanced the steroid ratio in urine (*P* = 0.0017) and kidney tissue (*P* < 0.0001), but not in plasma. When all the animals were taken as a group (*n* = 27), MAP increased with increasing urinary B/A ratio (*P* = 0.002, *r* = 0.54). Values are mean (±SEM), each group of animals comprised 6–9 animals.

**Figure 5:**
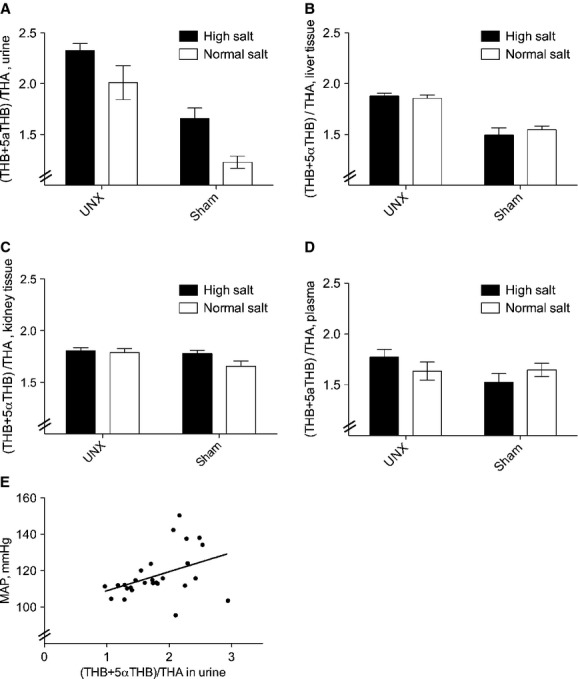
Comparison of (tetrahydrocorticosterone + 5α-tetrahydrocorticosterone)/tetrahydrodehydrocorticosterone (THB + 5αTHB)/THA in urine (A), liver tissue (B), kidney tissue (C), and plasma (D) between uninephrectomized (UNX) and sham-operated (Sham) rats fed a normal or high-salt diet. The ANOVA revealed that UNX increased the steroid ratios in urine and liver tissue (*P* < 0.0001) and as a tendency in kidney tissue (*P* = 0.0597), but not in plasma. A high-salt diet enhanced the urinary ratio in both sham and UNX rats (*P* = 0.0029). When all the animals were taken as a group (*n* = 27), MAP increased with increasing urinary (THB + 5αTHB)/THA ratio (*P* = 0.033, *r* = 0.41). Values are mean (±SEM), each group of animals comprised 6–9 animals.

The interconversions of the biologically active B to the inactive A are determined by the 11βHSD1 and 11βHSD2. An increased B/A ratio or (THA + 5αTHA)/THB ratio indicates either an increased 11βHSD1 and/or a decreased 11βHSD2 activity (Shackleton 1993). Figure 4 indicates that the B/A ratio in urine, plasma, and renal tissue increased after UNX in rats on a HS and on a NS diet. The increase of B/A in plasma and in the kidney, the organ with the quantitatively highest 11βHSD2 – activity within the mammalian body, was enhanced by HS diet (Fig. 4). With increasing B/A in urine the MAP increased. Similarly, MAP increased with urinary (THA + 5αTHA)/THB ratios (Fig. 5). The higher (THA + 5αTHA)/THB in urine after UNX is probably best explained by the hepatic tissue favouring an increased (THA + 5αTHA)/THB (Fig. 5B). A HS diet increased the urinary (THA + 5αTHA)/THB ratio but not those in the other matrices (Fig. 5). The mRNA for 11βHSD1 in liver tissue was slightly but not significantly higher after UNX and significantly increased by HS diet (Table 1).

## Discussion

In the present investigation, UNX induced salt-sensitive hypertension. The finding of salt sensitivity after UNX is in line with recent observations of Fujita et al. (2012). Interestingly, these authors could reverse the increase in blood pressure by the MR-antagonist eplerenone, but not by the aldosterone-synthase inhibitor FAD286 in rats on a HS diet after UNX. In the absence of a dose-finding study, one can not entirely exclude the possibility of underdosing of FAD286 as the cause of the absence of an effect. An alternative interpretation of their finding is the presence of an MR ligand other than aldosterone. The present observation of a diminished 11βHSD2 and an enhanced 11βHSD1 strongly supports the conclusion that the missing ligand accounting for the increase in blood pressure after UNX is an 11β-hydroxy-glucocorticoid (Fig. 1). The intracellular prereceptor concentrations of 11β-hydroxy-glucocorticoids are determined tissue specifically by the 11βHSD-enzymes (Edwards et al. 1988; Funder et al. 1988). The main site of MR action for blood pressure regulation is the cortical collecting duct. Therefore, ideally one should measure 11β-hydroxy-glucocorticoid concentrations directly in the cells of that tubular segment, what is not possible for technical reasons. Thus, as a surrogate for the activity of 11βHSD enzymes at the site of action, urinary glucocorticosteroid metabolites are considered (Shackleton 1993; White et al. 1997; Tomlinson et al. 2004). As shown in Figures 4 and 5, the apparent activities of 11βHSD2 and 11βHSD1 correlate with blood pressure.

The molecular mechanisms for changes in 11βHSD activities – not the primary purpose of the present investigation – remain by and large unresolved. The mRNA content of 11βHSD2 normalized by GAPDH in total kidney tissue diminished nonsignificantly from 0.112 to 0.075 in rats on a NS diet after UNX (Table 1), an observation congruent with a numerically similar, but significant decline from 0.11 to 0.08 in rats after UNX by Lauterburg et al. (2012) and in line with the diminished activity of 11βHSD2 in this study. In the study of Lauterburg, no rats on a HS diet were investigated; therefore, the effect of salt loading cannot be compared. Please note that the analysis of mRNA in total kidney tissue might not be an adequate strategy for measuring the transcriptional regulation in the biologically relevant target tissue, the cortical collecting duct. This might be particularly true in our model, where the kidney size after UNX increased dramatically, what was salient in rats on a HS diet (Fig. 3). If not all the tubular segments increased to the same extend after UNX and/or changing salt diet, then our measures of mRNA in total kidney tissue might be confounded by differential hyperplasia. To the best of our knowledge, there are no quantitative morphological measures about specific tubular length following UNX available from the literature. In a different model, Kaissling and Stanton (1988) showed in their seminal investigation that chronic furosemide and salt loading induced differential hyperplasia in the various tubular segments in rats.

The mRNA content of 11βHSD1 in the liver, a more homogenous organ than the kidney, increased after UNX in both, rats on a NS and a HS diet, a finding in line with the increase in the apparent 11βHSD1 activity. At variance, in the study of Lauterburg, both the hepatic activity and the mRNA of 11βHSD 1 declined in rats on a normal salt diet after UNX (Lauterburg et al. 2012). We have no other explanation for the different results than the different experimental conditions: the rats in this study were uninephrectomized at the age of 3 weeks and studied 10 weeks later, whereas those of Lauterburg were uninephrectomized at the age of 8 weeks and studied 5 weeks later.

The creatinine clearance per kidney and the weight of the remaining kidney after UNX increased in all rats when compared with the corresponding kidney in the sham-operated rats. The increase in weight of the remnant kidney in the NS fed rats is in line with the observation of Lauterburg et al. (2012). To our knowledge, the observation of a more pronounced increase in the weight of the remnant kidney in rats on a HS when compared with that on a NS diet is novel. The underlying mechanism and the consequences of this finding deserve further investigations.

While an increase in glucocorticoids in the target cells as a consequence of dysregulated 11βHSD enzymes has so far only been shown in artificial in vitro studies (Escher et al. 1997; Heiniger et al. 2001), there is an array of clinical and experimental evidence for their relevance in blood pressure regulation. Humans with a genetic or pharmacologically induced deficiency of 11βHSD2 exhibit increased blood pressure (Funder et al. 1988; Stewart et al. 1988; Ferrari et al. 2001; Frey et al. 2004; Atanasov et al. 2007). Similar observations were made in rodents including 11βHSD2-knockout mice (Kotelevtsev et al. 1999; Bailey et al. 2011; Fürstenberger et al. 2012) 11βHSD1 leads to hypertension (Masuzaki et al. 2003) and to a metabolic syndrome (Masuzaki et al. 2001), when overexpressed in adipose tissue in mice. These models appeared to increase blood pressure by elevating angiotensinogen levels and/or circulating glucocorticosteroid concentrations. The role of 11βHSD1 for hypertension is less clear in humans, although there is some evidence that a genotype that confers increased 11βHSD1 activity is associated with hypertension (Gambineri et al. 2011).

### Perspectives

There is little evidence that the effect and/or the regulation of the 11βHSD enzymes are different in rodents than in humans. Thus, one is tempted to speculate that antagonizing the MR with hypertension after UNX might be the first-line strategy in therapy in humans. Before such a conclusion can be applied, the apparent 11βHSD activities after UNX have to be studied and the side effects of the MR antagonist carefully investigated in humans. Practically more important might be to recommend subjects with one kidney, to adhere to a low-salt diet.
